# High flexion femoral side remnant preservation positioning technique: a new method for positioning the femoral tunnel in anterior cruciate ligament reconstruction

**DOI:** 10.1186/s13018-024-04670-7

**Published:** 2024-03-18

**Authors:** Xiaobo Li, Jiajun Lu, JIxian Su, Hanlin Li, Xiaoying Liu, Ran Ding

**Affiliations:** 1 Department of Orthopedics, General Hospital of Central Theater Command, 627 Wuluo Road, Wuchang District, Wuhan, Hubei Province China; 2 The First School of Clinical Medicine, Southern Medical University, Guangzhou, Guangdong Province China; 3 School of Medicine, Wuhan University of Science and Technology, 2 West Huangjiahu Road, Hongshan District, Wuhan, Hubei Province China; 4 Department of Spine, Trauma Surgery, The First People’s Hospital of Guangyuan, Guangyuan, Sichuan Province China; 5 Department of Respiratory and Critical Care Medicine, The First People’s Hospital of Guangyuan, Guangyuan, Sichuan Province China

**Keywords:** Anterior cruciate ligament (ACL) reconstruction, Remnant preservation, I.D.E.A.L. femoral tunnel, Three-dimensional CT

## Abstract

**Purpose:**

The aim of this study is to find a new method for femoral side preservation positioning in anterior cruciate ligament (ACL) reconstruction and test the accuracy and precision of this method.

**Method:**

Fifty patients with isolated ACL rupture (42 males and 8 females) who underwent single-bundle ACL reconstruction in our hospital between July 2022 and July 2023 were included. The lowest point of the cartilage margin of the lateral wall of the intercontinental fossa and the tibial plateau plumb line at 120° of knee flexion were used as the anatomical landmarks for positioning of the femoral tunnel for ACL reconstruction surgery. Femoral side remnant preservation was performed in all cases. Three-dimensional CT was performed 3 days postoperatively to collect the data, which were analyzed using Mimics 21.0 software. We measured the posterior cortical distance of the femoral condyle at 90° of knee flexion and the vertical distance from the center of the bone tunnel to the cortical extension line behind the femur. All femoral tunnel positions were marked on a 4 × 4 grid and visualized using the quadrant method.

**Results:**

Using the new positioning method in 50 knees, the average distance of *x* was 25.26 ± 2.76% of *t* and the average distance of *y* was 23.69 ± 6.19% of *h*. This is close to the results of previous studies, where *x* was 24.2 ± 4.0% of *t* and the average distance of *y* was 21.6 ± 5.2% of *h*. Most femoral tunnel positions were located in the same area. The D values were distributed as follows: 60% in the range of 0 to 2 mm, 24% in the range of 2 to 4 mm, and 16% more than 4 mm. The E values were distributed as follows: 80% in the range of 0 to 4 mm and 20% more than 4 mm.

**Conclusion:**

In arthroscopic ACL reconstruction, the knee was flexed at 120° and the lowest point of the cartilage edge of the lateral wall of the intercondylar fossa and the tibial plateau plumb line were used as anatomical landmarks for the positioning of the femoral bone tunnel, which resulted in more accurate femoral bone tunnel positioning, better reproducibility, and better preservation of the femoral stump compared to traditional positioning methods.

## Introduction

The anterior cruciate ligament (ACL) is an important structure for the stability of knee motion, and its tear is one of the most common knee joint injuries. Injuries to the ACL, which afflict more than 200,000 individuals yearly in the United States, account for more than half of all knee injuries [1]. The most important treatment method for ACL tears is ligament reconstruction [1, 2]. Approximately 130,000 ACL reconstruction surgeries are performed annually in the United States [3, 4]. Although positive outcomes of ACL reconstruction surgery have been reported, a review article indicates that a sizable minority of patients experience negative results and knee instability [5]. The effect of ACL reconstruction is affected by the location of the bone tunnel, the way of graft fixation, etc. A multicenter study of ACL revision reported that technique errors were the main cause of atraumatic ACL reconstruction failures, the majority of which were due to malpositioning of the tunnel socket [6].

The concept of ACL femoral bone tunnel positioning has gone through a process from isometric reconstruction to anatomical reconstruction. Biomechanical studies based on cadaveric specimens and clinical follow-up studies have found that isometric reconstruction is not effective in restoring knee rotational stability [7]. Therefore, anatomical reconstruction was favored at the beginning of the twenty-first century. Subsequent clinical studies demonstrated that anatomical reconstruction results in better rotational stability than isometric reconstruction, and isometric reconstruction of the footprint region has also been shown to have good isometric properties in a study by Forsythe et al. [8]. The clinical outcomes of anatomical single-bundle reconstruction and anatomical double-bundle reconstruction are still controversial, and most studies have concluded that there is no significant difference between anatomical double-bundle and anatomical single-bundle reconstruction of the ACL [9]. However, the anatomical single-bundle reconstruction technique is still the mainstream technique in clinical practice because of its advantage of simple operation. In recent years, with a deeper understanding of the histology and biomechanics of the ACL femoral stump, the concept of anatomical reconstruction has also made new advances. It has been claimed [10] that ACL single-bundle reconstruction centered on the anteromedial bundle results in better mechanical stability of the knee.

Existing methods of femoral tunnel localization, such as transtibial femoral offset guidance and the clock face technique, are inaccurate and outdated. Adam et al. [11] proposed to use the apex of the deep cartilage as an anatomical landmark to guide anatomical single-bundle ACL reconstruction. During surgery, clear visualization of the posterior cartilage margin of the lateral femoral condyle requires shaving the ACL femoral side stump cleanly, which loses the advantage of preserving the ACL stump. In recent years, authors such as Pearle et al. [12] have proposed the I.D.E.A.L. femoral tunnel, which refers to the placement of the femoral tunnel in a position that reproduces the isometry of the native ACL, covers the fibers of the direct insertion histologically, is eccentrically located in the anterior (high) and proximal (deep) regions of the footprint, anatomically (within the footprint), and replicates the low tension flexion pattern of the natural ACL throughout its range of flexion and extension. The idea of the I.D.E.A.L. femoral bone tunnel is now accepted by most scholars, but there is currently no consensus on the method of locating the I.D.E.A.L. femoral bone tunnel. A simple technique to assist in I.D.E.A.L. femoral tunnel positioning is therefore needed.

Femoral bone tunnel localization is traditionally performed with the knee joint flexed at 90°, but anatomical studies showed that at 90° of flexion, the socket of the posterior lateral bundle of the femoral ACL located anteriorly and inferiorly will obscure the femoral footprint area of the anteromedial bundle located posteriorly and superiorly. However, when the knee joint is flexed at 120°, due to the rotation of the femoral condyles, after the posterior lateral bundle moves anteriorly and superiorly, the anteromedial bundle moves anteriorly and inferiorly, and the latter is no longer obscured, which is more conducive to observation and localization. Therefore, by performing the procedure at 120° of knee flexion, better femoral socket exposure of the anteromedial bundle can be obtained without shaving off the posterior lateral femoral stump of the ACL. However, current studies [13–15] have mainly focused on the tibial side for stump preservation, and the femoral side is usually extensively cleaned to reveal the posterior cartilaginous margin of the lateral femoral condyle as an anatomical landmark. In the present study, the lowest point of the cartilage margin of the lateral wall of the intercondylar fossa of the knee and the tibial plateau drape were used as anatomical landmarks without revealing the posterior femoral wall, which can effectively preserve the femoral stump of the ACL. Thus, a simple and effective method of positioning the femoral bone tunnel that preserves the femoral side remnant of the ACL was explored.

The purpose of the present study was to investigate the use of the lowest point of the cartilaginous margin of the lateral femoral condyle and the tibial plateau as anatomical landmarks at 120° of knee flexion to guide femoral tunnel placement during anatomical single-bundle ACL reconstruction. This method can be used intraoperatively for arthroscopy.

## Materials and methods

All procedures were performed by the same experienced surgeon, who performs more than 200 ACL reconstruction procedures per year. Fifty patients with unilateral ACL injuries who underwent primary single-bundle ACL reconstruction between July 2022 and December 2022 were included in this study (42 male and 8 female patients).

Inclusion criteria were as follows: the patient had a clear history of injury and instability in the affected knee; complete ACL rupture confirmed by preoperative magnetic resonance imaging (MRI) and intraoperative arthroscopy; initial ACL reconstruction; no surgical or invasive operations had previously been performed on either knee of the patient; all participants provided informed consent.

Exclusion criteria were as follows: revision ACL reconstruction, reconstruction of multiple ligament injuries, joint allograft meniscus graft, contralateral knee ACL reconstruction, combined severe cartilage injury, combined intercondylar fracture, tibial plateau fracture, etc. ACL reconstruction was performed using an autologous hamstring graft (including semitendinosus and gracilis).

The diameter of the tendon graft was kept between 8 and 9 mm [16]. The tibial bone graft was fixed with an absorbable interfacial extrusion screw (Smith & Nephew Inc, USA), while the femoral end was fixed with an Endobutton belt loop titanium plate (Smith & Nephew Inc, USA). The transpotal technique was used when drilling the femoral tunnel, as the postoperative ACL angle on the MRI of the knee is closer to the healthy side [17]. The type of ACL rupture was determined during the arthroscopic diagnosis and a decision was made whether to preserve the ACL stump. The following are some of the possible benefits of preserving the ACL stump [2, 18]: it orients native collagen in the direction of the ACL graft; advantages of standard anatomical ACL reconstruction are not lost; no loose stump in the notch; cyclops lesions are prevented; better healing and ligamentization; and no additional implants are needed.

### Graft harvest and preparation

**Fig. 1:**
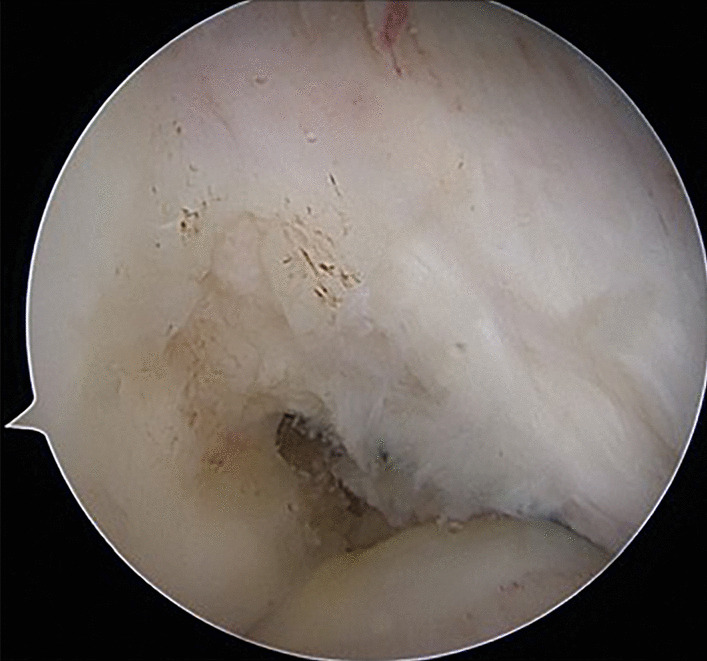
Arthroscopic ACL: partial tear of the ACL, femoral side stump of the ACL

Standard anteromedial and anterolateral incisions were made. The patient was placed in the supine position and epidural or general anesthesia was administered. The anteromedial approach was used for exploration and the anterolateral approach was used for standard arthroscopy. At 90° of flexion, the synovial membrane and part of the infrapatellar fat pad were removed to fully expose the medial anterior aspect of the lateral femoral condyle for easy visualization and localization. ACL rupture was confirmed (Fig. 1), and the arthroscope was withdrawn. A 2-cm skin incision was made superiorly on the medial tibia to reveal the goose foot tendon. The semitendinosus and gracilis were then removed with a tendon retriever.

### Femoral tunnel preparation

**Fig. 2:**
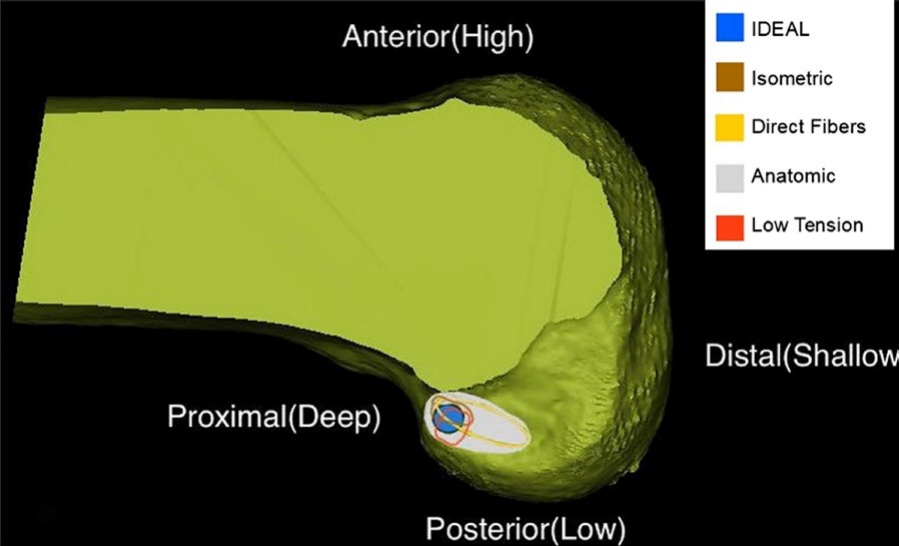
The blue circle (I.D.E.A.L. femoral tunnel) indicates the ideal placement of the femoral tunnel in single-bundle ACL reconstruction

**Fig. 3:**
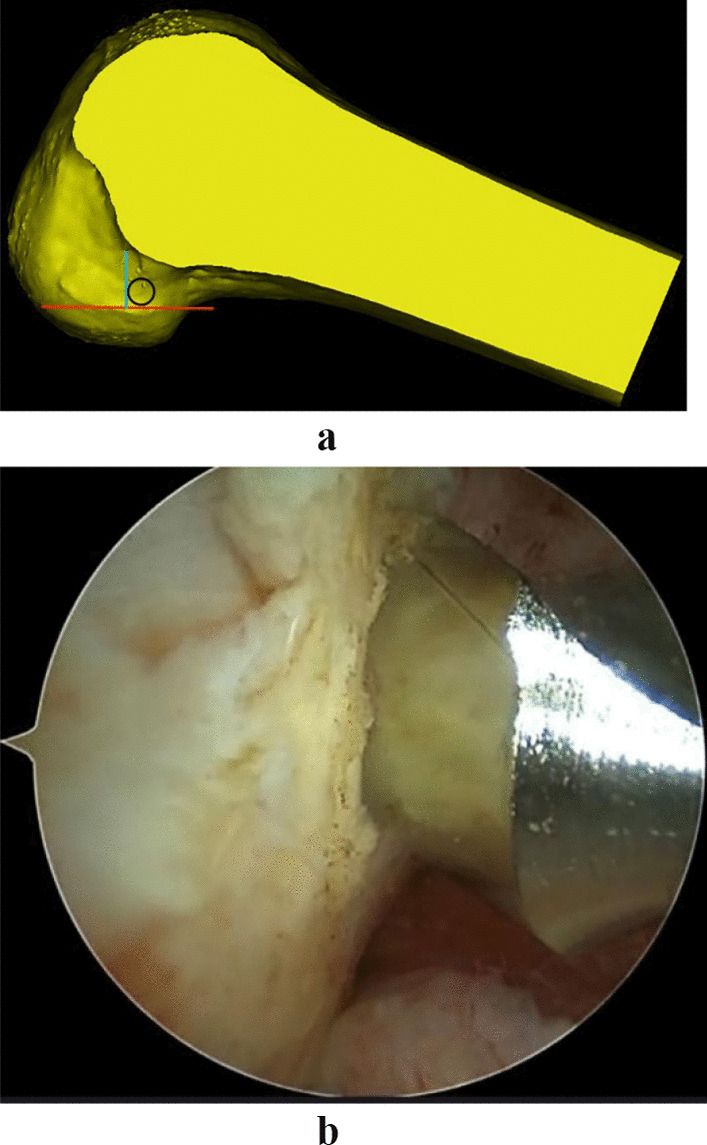
**a** Red line: the lowest point of the cartilage margin horizontal line. Blue line: the vertical line of the red line. Black circle: circle tangent to the red and blue lines (hollow drill). **b** Arthroscopic view

Most surgeons accept the theory of I.D.E.A.L. femoral location (Fig. 2). The I.D.E.A.L. location is located a little below the footprint of the direct fiber insertion of the anteromedial ACL bundle.The flexion mode arthroscopic femoral tunnel is positioned by first flexing the knee 120° using the anterolateral approach as the observation approach and identifying the lowest point of the cartilage margin of the femoral condyle, through which a vertical line is made with a plasma device perpendicular to the tibial plateau, which is the bifurcate ridge between the anteromedial bundle and the posterolateral bundle. A hollow drill with the same diameter as the weave was selected. The hollow drill was introduced through an anterolateral medial inferior approach in the following position: the anterior edge of the drill was tangential to the vertical line, and the underside of the drill was located approximately 2 mm from the edge of the femoral condylar cartilage. The hollow drill was used to guide a 2 mm K wire and drill through the lateral femoral cortex. Then a fine bone tunnel was drilled with a 4.5 mm hollow drill, and the depth of the tunnel was measured; according to the length of the bone tunnel and the length of the graft, a coarse bone tunnel with suitable depth was created. This is called the high flexion femur preservation and residual positioning method (Figs. 3a and b).

### Tibial tunnel preparation and fixation of the graft

**Fig. 4:**
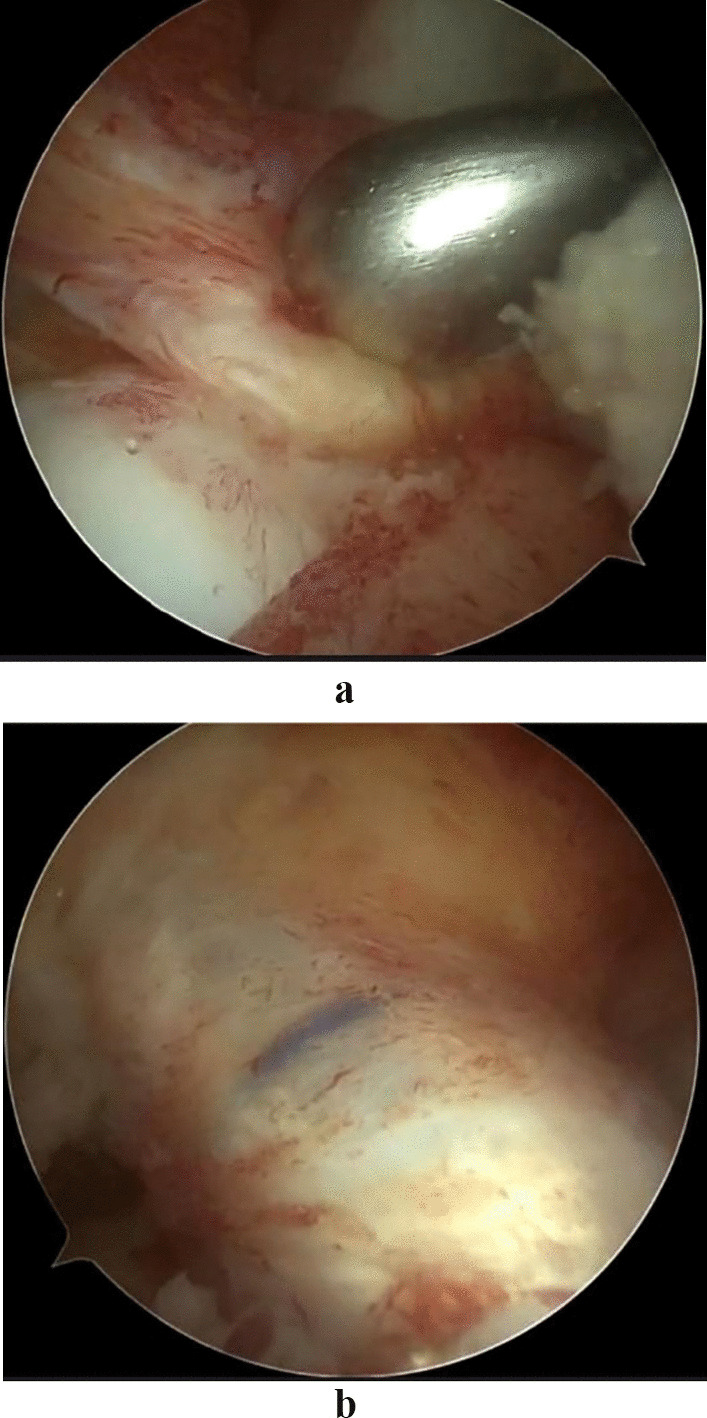
**a** Arthroscopic ACL reconstruction. Care must be taken to protect the ACL stump when drilling the tibial tunnel. **b** In ACL reconstruction, the graft is completely covered by the stump

The tibial locator was placed in the center of the ACL stump through the anteromedial entrance, then the tibia was drilled with a 2 mm K wire, and finally, the appropriate tibial drill was selected according to the diameter of the tendon. Finally, the tendon was introduced into the bone tunnel, and it was confirmed that the Endobutton was turned and fit well at the outer opening of the bone tunnel. Arthroscopic examination confirmed that the previous markings on the graft were flush with the entrance to the femoral bone tunnel, indicating complete entry of the graft into the femoral tunnel. The squeeze screw was placed at 15° of knee flexion to maintain pressure on the graft when the reconstructed ligament tension was closest to the original ACL tension [19]. Graft impingement was assessed during the procedure with the knee fully extended (Figs. 4a and b).

### Measurements

**Fig. 5:**
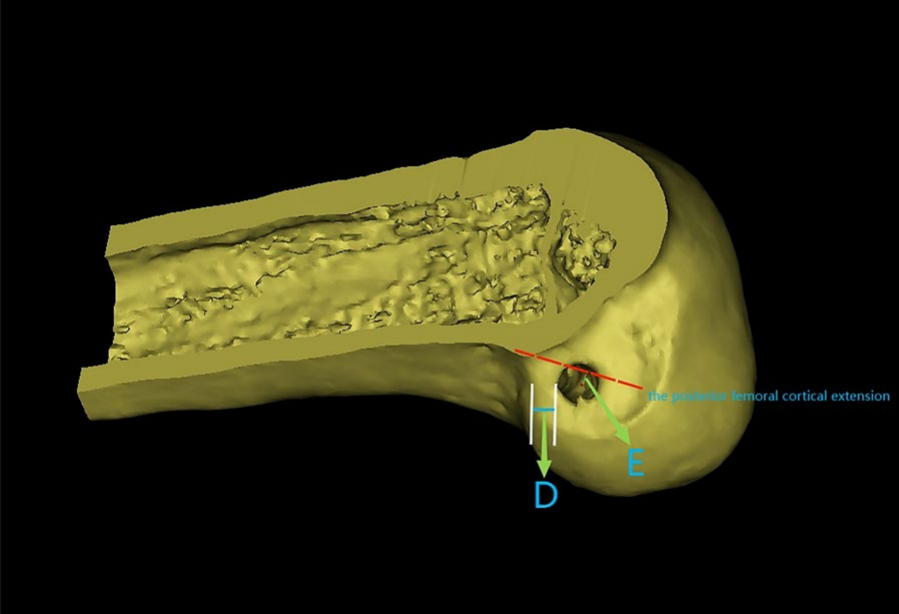
D represents the thickness of the posterior wall of the bone tunnel, E represents the distance of the center of the bone tunnel from the posterior femoral cortical extension, the red dashed line represents the posterior femoral cortical extension (intercondylar ridge), and the red dot represents the center point of the bone tunnel

**Fig. 6:**
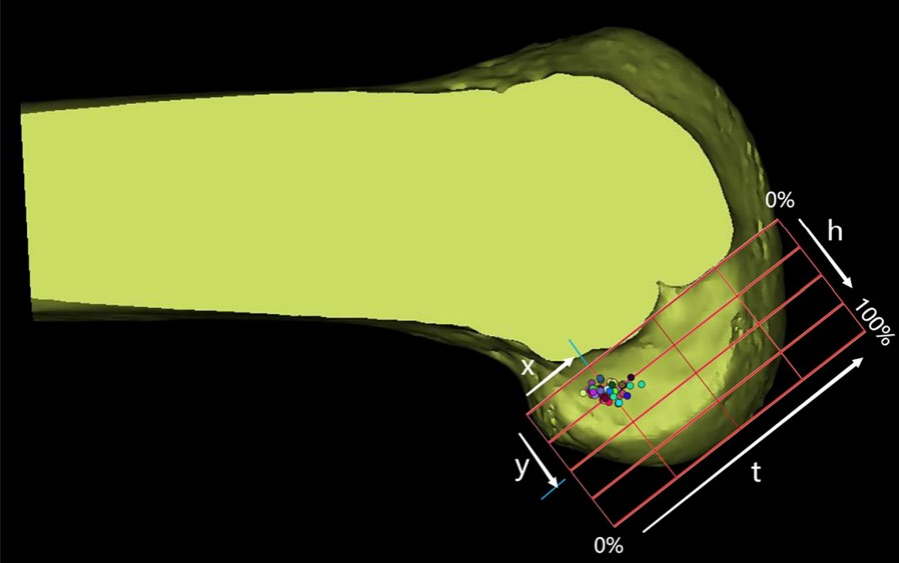
$x$, distance from the center of the footprint to the proximal border along line $t$; $y$, distance from the center of the footprint to the Blumensaat line; $x$ and $y$ are expressed as percentages of $t$ and $h$. $t$, total sagittal diameter of lateral condyle along the Blumensaat line; $h$, maximum intercondylar notch height.

Three days after the operation, all patients underwent 3D CT in a knee extension position. The Digital Imaging and Communications Medicine (DICOM) data (Siemens 64-layer spiral CT, 1 mm thick) of the patients' postoperative knee CT images were imported into Mimics 21.0 software (Materialise, Leuven, Belgium) to reconstruct knee models (distal femur and proximal tibia), which were used to assess the position of the femoral tract of the ACL. The sagittal cut surface of the knee can be constructed as follows: first, the condylar axis is determined and the posterior and distal femoral condyles are approximated cylindrically to provide a true lateral image of the femur. This is because the intercondylar notch is different for each individual, so the axial length of the condylar axis is fixed at 100%. We used the same cutting plane for all reconstructed knee joints according to a related study [20]: at the apex of the intercondylar notch, we established a single cutting plane perpendicular to this condylar axis (C-plane). After resection of the medial femoral condyle in the C-plane, an image of the lateral femoral condyle in true lateral position was obtained. This view was used to measure the position of the femoral tunnel. The cortical extension line behind the femur was approximately regarded as the direct fiber insertion point of the ACL [21, 22]. The data were merged using Bernard and Hertel's quadrant approach [23], which has been widely used for evaluating the position of the ACL femoral footprint [23]. In the quadrant approach, there are four distances: the total sagittal diameter of the lateral condyle along the Blumensaat line (distance *t*), the maximum intercondylar notch height perpendicular to the Blumensaat line (distance *h*), the distance from the center of the footprint to the proximal border along line *t* (distance *x*), and the distance between the Blumensaat line and the center of the footprint (distance *y*). The distances *x* and *y* are expressed as percentages of *t* and *h*, respectively. All femoral bone tunnels were marked on a 4 × 4 grid, and the quadrant method was used to visualize the area of these tunnels. Next, dots were created to show the centers of all the bone tunnels using Photoshop (Fig. 6). The distance from the center of the circle to the *t* and *h* axes was measured as the location of the femoral bone tunnels. Finally, we utilized the sagittal plane of the lateral condyle of the knee obtained from the Mimics software for the measurement of D and E values (Fig. 5). The D value represents the thickness of the posterior wall of the bone tunnel; the E value represents the distance of the center of the bone tunnel from the posterior femoral cortical extension. The D and E values are represented as a scatterplot. The data for the location of the femoral tunnel centers are expressed as mean and standard deviation. Data were measured twice by the same surgeon at intervals greater than 1 week.

### Statistical analysis

**Fig. 7:**
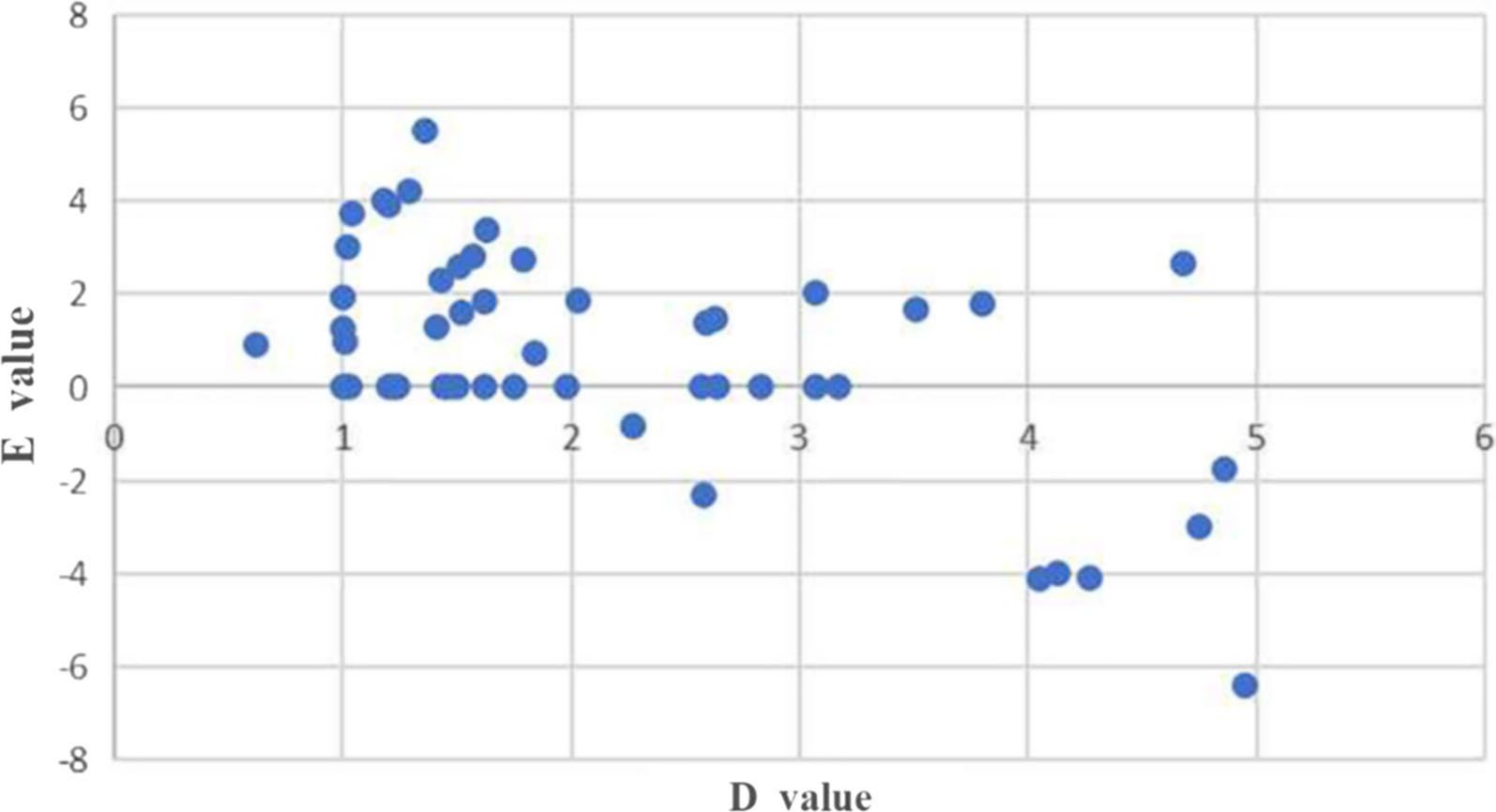
Scatterplot of D and E values

Statistical analysis was carried out using SPSS software version 25.0 (SPSS Inc., Chicago, IL, USA). The mean and standard deviation were calculated. Scatterplots of D and E values were generated using Microsoft Excel 2016 (Fig. 7).

## Results

As shown in Fig. 6, with the new localization method, the average distance *x* was 25.26 ± 2.76% of *t* and the average distance of *y* was 23.69 ± 6.19% of *h.* As shown in Fig. 7, the D values were distributed as follows: 60% in the range of 0 to 2 mm, 24% in the range of 2 to 4 mm, and 16% more than 4 mm. The E values were distributed as follows: 80% in the range of 0 to 4 mm and 20% more than 4 mm. Our results are in agreement with Table 1, which was obtained from Xu et al. [24]. Eight studies have shown that the theoretical center of the anteromedial bundle is 24.2 ± 4.0 × 21.6 ± 5.2. Based on Mimics measurements, these results are close to the location of the I.D.E.A.L. point reported in the present study. We believe that with the new localization method, our femoral tract is very close to the I.D.E.A.L. point. Therefore, the new positioning method can be used as a simple method to place the I.D.E.A.L. femoral tunnel in clinical ACL reconstruction and significantly shorten the operation time.

**Table 1:** Anatomic center of ACL femoral footprint

No.	Article (year)	Center of anteromedial bundle, %, $x$ × $y$, M ± SD	Number of cases	Method for measurement
1	Yamamoto (2004) [43]	25.0 ± 5 × 16.0 ± 5	10	Standard lateral radiographs
2	Colombet (2006) [44]	26.4 ± 2.6 × 25.3 ± 4.2	7	Standard lateral radiographs
3	Tsukada (2008) [45]	25.9 ± 2 × 17.8 ± 2.9	36	Standard lateral radiographs
4	Lorenz (2009) [46]	21 ± 3 × 22 ± 2	12	Standard lateral radiographs
5	Forsythe (2010) [47]	21.7 ± 2.5 × 33.2 ± 5.6	8	3D CT, similar to standard lateral radiographs position
6	Iriuchishima (2010) [48]	15 ± 6 × 26 ± 8	15	Standard lateral radiographs
7	Pietrini (2011) [49]	21.6 ± 5.6 × 14.2 ± 7.7	12	Standard lateral radiographs
8	Zantop (2008) [50]	18.5 × 22.3	20	Standard lateral radiographs
	Theoretical center* (pooled M ± SD)	24.2 ± 4.0 × 21.6 ± 5.2	–	–

$x$, distance from the center of the footprint to the proximal border along line $t$; $y$, distance from the center of the footprint to the Blumensaat line; $x$ and $y$ are expressed as percentages of $t$ and $h$. $t$, total sagittal diameter of the lateral condyle along the Blumensaat line; $h$, maximum intercondylar notch height; CT, computed tomography; M ± SD, mean ± standard deviation.

*The theoretical center of the anteromedial bundle was deduced by the pooled M ± SD of eight studies.

## Discussion

A variety of factors have an impact on the outcome of ACL reconstruction surgery, and we are still searching for the ideal approach. The clinical results of stump-preserving reconstruction with an I.D.E.A.L. femoral tunnel have been documented in previous clinical studies [2]. The anteromedial and posterolateral bundles of the ACL have different functions. However, biomechanical studies have shown that the anteromedial bundle is the main factor associated with knee stability [25]. Several recent studies have shown that tunnel reconstruction in the center of the anteromedial bundle is desirable [26, 27]. In the present study, we performed anteromedial bundle reconstruction of the ACL.

In the present study, we performed the operation with the knee joint flexed at 120°, and the lowest point of the cartilaginous margin of the lateral femoral condyle (at 120° of flexion) and the tibial plateau were used arthroscopically as anatomical landmarks. In this way, we were able to obtain better femoral side exposure of the anteromedial bundle without extensively cleaning the posterior lateral femoral bundle stump of the ACL, and we could achieve better stump-preserving reconstruction of the femoral side of the ACL. Recent studies of residual preservation have mainly focused on the tibial side, and the femoral side is usually extensively cleaned to reveal the posterior femoral wall as an anatomical landmark. In arthroscopic surgery, removal of the ACL stump helps to improve visualization of bony landmarks on the tibia and femur and helps to localize the bone tunnel, thus making the procedure easier.

From the eight studies in Table 1, we can see that the center of the anteromedial bundle of the ACL that was ultimately derived was 24.2 ± 4.0 × 21.6 ± 5.2, whereas the center of the anteromedial bundle of the ACL in our present study was 25.26 ± 2.76 × 23.69 ± 6.19. In the present study, in 84% of the cases, the posterior wall of the femoral bone tunnel was maintained at 0–4 mm, of which 0–2 mm accounted for 60%, and our femoral bone tunnel position was very close to the posterior cartilaginous margin of the lateral condyle of the femur. The high flexion femoral side remnant preservation positioning technique is safe and avoids breaking the posterior wall of the femoral osseous tract. This is similar to the findings of authors such as Smith et al. [28], who placed the femoral bone tunnel less than 2 mm posterior to the posterior wall of the bone tunnel. In the present study, there was not one case of posterior wall rupture of the femoral bone tunnel, which indicates the safety of our high flexion femoral side remnant preservation positioning technique. Moreover, we created the femoral bone tunnel with the knee kept in flexion at 120° during the procedure, which also reduced the risk of posterior wall rupture to a certain extent. Chung et al. [29] indicated that creating the femoral osseous tract by keeping the knee flexion angle between 120° and 130° can avoid posterior wall rupture and achieve an appropriate femoral channel length.

The role of stump preservation in ACL reconstruction has been extensively studied. However, preserving the ACL stump has its drawbacks. Preservation of the stump has been shown to be a risk factor for arthrofibrosis and the formation of cyclops lesions in the knee joint.

We believe that a new method is needed to ensure the correct placement of the femoral tunnel. In the present study, the lowest point of the cartilage margin of the lateral wall of the intercondylar fossa of the knee and the vertical line between the tibial plateau and the lowest point were used as anatomical landmarks to create the ACL femoral bone tunnel at 120° of knee flexion, which enables the reconstruction of the preserved femoral stump while accurately positioning the femoral bone tunnel.

There are some limitations to this study. First, only one surgeon confirmed the location of the femoral bone tunnel during ACL reconstruction. There is currently no golden standard for the intraoperative localization of the I.D.E.A.L. femoral bone tunnel. Second, the number of cases in our study was small. For subsequent studies, we aim to include more patients to confirm our conclusions. Finally, there are anatomical differences in the knee joint that may lead to incorrect positioning.

## Conclusions

In this study, during ACL reconstruction, the lowest point of the cartilage margin of the lateral wall of the intercondylar fossa of the knee and the vertical line between the tibial plateau and the lowest point were used as anatomical landmarks, and the ACL femoral tunnel was established at 120° of knee flexion, which could achieve stump-preserving reconstruction of the femur while accurately locating the femoral bone tunnel. This technique would not sacrifice the ideal position of the femoral tunnel and is able to retain the possible benefits of the ACL stump.

## Data Availability

The datasets used or analyzed during the current study are available from the corresponding author upon reasonable request.
